# Evaluation of the antibacterial, antibiofilm, and anti-virulence effects of acetic acid and the related mechanisms on colistin-resistant *Pseudomonas aeruginosa*

**DOI:** 10.1186/s12866-022-02716-6

**Published:** 2022-12-19

**Authors:** Luozhu Feng, Mengxin Xu, Weiliang Zeng, Xiaodong Zhang, Sipei Wang, Zhuocheng Yao, Tieli Zhou, Shiyi Shi, Jianming Cao, Lijiang Chen

**Affiliations:** 1 Department of Clinical Laboratory, The First Affiliated Hospital of Wenzhou Medical University; Key Laboratory of Clinical Laboratory Diagnosis and Translational Research of Zhejiang Province, Wenzhou, Zhejiang Province China; 2 Department of Medical Lab Science, School of Laboratory Medicine and Life Science, Wenzhou Medical University, Wenzhou, Zhejiang Province China

**Keywords:** Acetic acid, Colistin-resistant *P. aeruginosa*, Antibacterial, Antibiofilm, Antivirulence, Mechanisms

## Abstract

**Background:**

*Pseudomonas aeruginosa* (*P. aeruginosa*) has been majorly implicated in the infection of burns, wounds, skin, and respiratory tract. Colistin is considered the last line of defense against *P. aeruginosa* infections. However, colistin is becoming increasingly invalid in treating patients infected with colistin-resistant (COL-R) *P. aeruginosa*. As one of the disinfectants used for wound infections, acetic acid (AA) offers good antibacterial and antibiofilm activities against *P. aeruginosa*. This study investigated the effects of AA on COL-R *P. aeruginosa* in terms of its antibacterial, antibiofilm, and anti-virulence properties and the corresponding underlying mechanisms.

**Results:**

The antimicrobial susceptibility and growth curve data revealed that 0.078% (v/v) AA exhibited good antibacterial activity against COL-R *P. aeruginosa*. Subinhibitory concentrations of AA were ineffective in inhibiting biofilm formation, but 4 × and 8 × of the minimum inhibitory concentration (MIC) was effective in removing the preformed biofilms in biofilm-eradication assays. The virulence results illustrated that AA inhibited COL-R *P. aeruginosa* swimming, swarming, twitching, and pyocyanin and elastase production. The analysis of the potential antibacterial mechanisms of AA on COL-R *P. aeruginosa* revealed that AA acted by increasing the outer and inner membrane permeability, polarizing the membrane potential, and decreasing the reduction potential in a concentration-dependent manner. The qRT-PCR results revealed that AA may inhibit the virulence of COL-R *P. aeruginosa* by inhibiting the expression of T3SS-related and QS-related genes.

**Conclusions:**

AA possesses antibacterial, antibiofilm, and anti-virulence properties that ultimately lead to the alteration of the bacterial membrane permeability, membrane potential, and reduction potential. Our findings indicated that AA is presently one of the effective treatment options for infections. A high concentration of AA (> 0.156% v/v) can be used to sterilize biofilm-prone surgical instruments, for hospital disinfection, and for treating the external wound, whereas a low concentration of AA (0.00975–0.039% v/v) may be used as an anti-virulence agent for adjuvant treatment of COL-R *P. aeruginosa*, thereby further improving the application value of AA in the treatment of infections.

**Supplementary Information:**

The online version contains supplementary material available at 10.1186/s12866-022-02716-6.

## Background

*Pseudomonas aeruginosa* (*P. aeruginosa*) is a gram-negative pathogen that causes infections in the eyes, ear, skin, urethra, blood, and respiratory tract in burn patients and other immunocompromised individuals [1]*. P. aeruginosa* can structure colonies of bacteria contained in a protective polysaccharide matrix, prevent re-epithelization, stimulate chronic inflammation, and protect it from endogenous and exogenous antimicrobial drugs [2], which together pose a major challenge in clinical anti-infective treatment [3]. Furthermore, virulence is a concern, especially in cases of acute infections as *P. aeruginosa* possesses an intrinsic ability to produce a myriad of virulence factors, such as flagella, type IV pili, pyocyanin, elastase, alkaline phosphatase, and rhamnolipids [4]. Vanderwoude et al. reported the nature of virulence evolution during chronic infection within-host adaptation that resulted in higher virulence depending on the setting [5]. In addition, *P. aeruginosa* has been included in the “critical” category of the World Health Organization’s (WHO) priority list of bacterial pathogens [6], which makes it urgent and important to undertake research and development activities for new antibiotics against *P. aeruginosa* infections.

Colistin was reintroduced in medical therapy as the last-resort treatment in several infections caused by multi-drug resistant (MDR) and extensively drug-resistant (XDR) *P. aeruginosa* [7]. However, with the widespread use of colistin, the emergence of colistin-resistant (COL-R) *P. aeruginosa* is increasing. Innovative alternative approaches to controlling and avoiding COL-R *P. aeruginosa* infection are required.

Acetic acid (AA), topical antiseptics, and disinfectants have been reported to be effective in otitis externa [8], burn or soft tissue wounds [9], and purulent bronchiectasis [10]. In addition, AA is used to eradicate biofilms from tympanostomy tubes [11]. The US Food and Drug Administration has approved a 0.25% AA solution for bladder irrigation and a 2% solution for treating external otitis [12]. Other studies have shown that 1% AA eliminates MDR *P. aeruginosa* in chronic wounds that have not responded to conventional therapies, including oral or injectable antibiotics and local wound care with hydrogen peroxide and betadine [13]. Bjarnsholt et al. reported pH of AA below 4.76 was effective against *P. aeruginosa* biofilms [14]. Since AA has good antibacterial and biofilm activity against *P. aeruginosa*, there are few reports on whether it possesses antibacterial, antibiofilm, and anti-virulence potential against COL-R *P. aeruginosa*. Therefore, this study aims to explore the effects of AA on COL-R *P. aeruginosa* derived from sputum and wounds in antibacterial, antibiofilm, anti-virulence, and mechanisms to improve the use of AA in the treatment of infections.

## Methods

### Bacterial strains and chemicals

A total of 8 non-duplicated clinical COL-R *P. aeruginosa* were recovered from the First Affiliated Hospital of Wenzhou Medical University in China. Among them, except for TL1671 derived from trauma seepage surface, the other 7 COL-R *P. aeruginosa* strains were isolated from the sputum surface. These strains were all identified by matrix-assisted laser desorption/ionization time-of-flight mass spectrometry (MALDI-TOF/MS; bioMérieux, Lyon, France). *P. aeruginosa* ATCC27853 was used as the control. Colistin and AA were purchased from Wenzhou Kangtai Biological Technology Co., Ltd (Zhejiang, China) and Sigma-Aldrich (Saint Louis, USA), respectively.

### Antimicrobial susceptibility test

The minimum inhibitory concentration (MIC) of colistin and AA were determined by using the cationic adjusted Mueller–Hinton broth (CAMHB) microdilution method [15]. Respectively, colistin or AA was diluted twice and subjected to serial two-fold dilutions from 128 to 0.0625 μg/mL for colistin and 5–0.005% (v/v) for AA prepared in CAMHB 96-well microtiter plates. A final bacterial suspension of 1.5 × 10^6^ CFU/mL was added to each well, and the plate was incubated with colistin or AA at 37℃ for 18 h. The interpretation of the antimicrobial susceptibility test was based on the breakpoint point of antibiotics provided by 2020 CLSI (intermediate ≤ 2 μg/mL; resistant ≥ 4 μg/mL), with each MIC test verified as a duplicate [16].

### Bacterial growth monitoring

To assess the effect of AA on COL-R *P. aeruginosa* growth, the bacterial cultures were grown in the absence or presence of AA at 37℃ for 24 h, followed by measurement of absorbance at 600 nm every 2 h. The bacterial growth curve for untreated cultures (control condition) and treated cultures was determined by plotting the values against time [17].

### Biofilm-formation inhibition assay

*P. aeruginosa* was cultured overnight on Columbia blood plates. The bacterial suspension was prepared and adjusted to 0.5 McFarland and diluted 1:100 in fresh Luria–Bertani (LB) broth. The suspension was then spread on 96-well plates and cultured overnight in the absence or presence of AA at 37℃ for 24 h*.* The culture supernatant was discarded after incubation. The plates were washed thrice with water to remove any remaining planktonic cells. Biofilms formed on the plates were stained for 15 min with 1% crystal violet, the excess dye was removed by washing thrice, and the bound crystal violet was solubilized in 95% ethanol. The absorbances were measured at 600 nm [18].

### Biofilm eradication assays

The removal of preformed biofilms by using AA was performed as described earlier [19]. Briefly, the bacterial suspension prepared in LB broth was added to a 96-well microtiter plate and incubated for 24 h for biofilm formation. Then, the planktonic cells were removed after incubation. The established biofilm cells were treated with or without AA within a fresh LB medium. Then, the microtiter plate was incubated at 37 °C for 24 h, and the biofilm cells were quantified after staining with 0.1% crystal violet according to the procedure described previously [20]. The experiments were conducted in triplicate. *p* < 0.05 was considered to indicate statistical significance.

### Motility assay

*P. aeruginosa* has a single polar flagellum and type-IV pili that enables them to swim in low-agar (< 0.4%) medium and propagate at the surface interfaces. In addition to swimming and twitching, *P. aeruginosa* can propagate on semisolid surfaces (i.e., 0.4–1.0% agar) in a coordinated manner through swarming motility [21]. The effect of AA on COL-R *P. aeruginosa* swimming, swarming, and twitching motilities were determined as described previously [22]. To monitor the swimming activity, 2 μL of the bacterial suspension was cultured overnight and spotted onto plates containing 0.3% (w/v) Bacto agar, 0.2% casamino acids (w/v), and 30 mM glucose in the presence or absence of subinhibitory concentrations (1/2 × MIC, 1/4 × MIC, 1/8 × MIC, 1/16 × MIC) of AA or hydrochloric acid (HA) corresponding to the pH of each concentration of AA, followed by incubation for 24 h at 37 °C. To monitor the swarming activities, 2 μL of the bacterial suspension cultured overnight was spotted onto the centers of treated and untreated swarming plates composed of 0.4% (w/v) Bacto agar and LB supplemented with 0.5% (w/v) casamino acids and 0.5% (w/v) glucose, followed by incubation at 37 °C for 24 h. For the twitching assay, *P. aeruginosa* was inoculated onto the bottom of a Petri dish containing subinhibitory concentrations (1/2 × , 1/4 × , 1/8 × , 1/16 × MIC) of AA or HA corresponding to the pH of each concentration of AA by stabbing a toothpick through a 2-mm thin layer of LB medium supplemented with 0.2% casamino acids, 30 mM glucose, and 1.5% Bacto agar. After incubation for 24 h at 37 °C, the agar was gently removed, and the Petri dish was air-dried. Then, 1% crystal violet solution was used to stain the plate agar interface for 10 min. Finally, the Petri dish was rinsed, and the crystal violet-stained twitching pattern was evaluated. The migration distance around the incubation site was also measured. The migration distance was found to be directly proportional to the motility ability.

### Pyocyanin assay

*P. aeruginosa* produces a secondary metabolite, pyocyanin, which can change the level of intracellular redox and induce oxidative damage to the host, making it one of the main causes of death in patients infected with *P. aeruginosa* [23]. The effect of AA on pyocyanin production was measured as described previously [24]. *P. aeruginosa* cultures were grown for 16–20 h in the presence or absence of subinhibitory concentrations (1/2 × , 1/4 × , 1/8 × , 1/16 × MIC) of AA. The pyocyanin concentration was estimated by vortexing 7.5 mL of filtered supernatant with 4.5 mL of chloroform until the color changed to greenish blue. The samples were then centrifuged (10,000 × *g* for 10 min), and 3 mL of the resultant blue-colored liquid was transferred into a fresh tube containing 1.5 mL of 0.2 M HCl and agitated until the blue color changed to pink. The absorbance of the pink layer was measured at 520 nm after it was transferred into a cuvette [25]**.** The absorbance obtained was proportional to the pyocyanin content.

### Elastase assay

Elastase is a protease secreted by *P. aeruginosa* that interacts with the host during pathogen infection and plays a key role in invasiveness [26]. The effect of AA on elastase production by *P. aeruginosa* was measured as described elsewhere [27]. Briefly, *P. aeruginosa* cultures were grown for 16–20 h with subinhibitory concentrations (1/2 × , 1/4 × , 1/8 × , 1/16 × MIC) of AA or no AA (control). Filtered supernatants were mixed in the ratio 2:1 with phosphate buffer (0.1 M, pH 6.3) and 2 mg/mL elastin Congo red (Sigma). For 24 h, the mixture was incubated at 37℃ under 200 rpm shaking. A spectrophotometer zeroed on an elastin Congo red sample cultured with medium alone was used to determine the absorbance of the supernatant at 495 nm after centrifugation [28]. The absorbance was proportional to the elastase content.

### Cell viability assay

RAW 264.7 macrophages (5 × 10^4^ macrophages in 100 μL of complete DMEM) were seeded in a 96-well tissue culture plate and incubated at 37℃ under a 5% CO_2_ atmosphere. After 12 h of incubation, 10 μL of AA prepared in a series of concentrations (0.312%, 0.156%, 0.078%, 0.039%, 0.0195%, and 0.010% were added to the cell culture medium. *P. aeruginosa* supernatant treated with or without AA was added to the cell culture media. As a control, LB media was added to the cell culture media and incubated for 12 h. After 12 h, the cells cultured with the tested compounds were washed with phosphate-buffered saline (PBS) and incubated for 4 h in 100 μL of 10% CCK8 solution. The absorbance of the converted dye in living cells was measured at the wavelength of 450 nm [29].

### Resazurin assays

The reducing potential was determined with Resazurin (PrestoBlue; ThermoFisher Scientific, Waltham, Massachusetts, USA), wherein resazurin was reduced by the cell metabolic activity. The mid-log-phase cells were transplanted into a black polystyrene 96-well plate containing varied concentrations (1 × , 1/2 × , 1/4 × MIC) of AA. AA (prepared in a series of concentrations) was added, and the plate was incubated with shaking for 30 min. Then, 20 μL Resazurin was added to the plate 5 min before the indicated time point [30]. The plate was then incubated with shaking in the dark at room temperature for 5 min. Finally, fluorescence readings were taken (excitation wavelength[ex], 570 nm/emission wavelength[em], 650 nm) using the BioTek Synergy H1 plate reader [29].

### Outer membrane permeability assay

Outer membrane permeability was assessed by the 1-N-phenylnaphthylamine (NPN) uptake assay [31]. The fluorescence of this probe increases when incorporated into the hydrophobic core of a permeabilized outer membrane. A graded series of AA concentrations was prepared (1 × , 1/2 × , 1/4 × , 1/8 × , 1/16 × MIC). HA corresponding to the pH of each concentration of AA and colistin (2 μg/mL) served as the positive control was added to mid-log-phase cells. The cultures were then sampled at 2 h. The cells were pelleted, washed twice, and resuspended in PBS. Finally, the NPN solution (final concentration, 30 μM) was added to the cells and incubated at 37 °C for 30 min. The fluorescence was immediately measured using the BioTek Synergy H1 plate reader (λexc/λem: 340/410 nm).

### Inner membrane permeability assays

To measure the disruption of the inner membrane barrier function, the membrane integrity dye propidium iodide (PI) was used. PI is a nucleic acid dye that cannot pass through normal membranes owing to their barrier action, but it can dye the nucleus red in necrotic cells with altered membrane permeability [32]. A graded series of AA concentrations (1 × , 1/2 × , 1/4 × , 1/8 × , 1/16 × MIC) were prepared. HA corresponding to the pH of each concentration of AA and colistin (2 μg/mL) served as the positive control was added to mid-log-phase cells. The cultures were sampled at 2 h. The cells were pelleted, washed twice, and resuspended in PBS. Finally, PI (50 μg/mL, Life Technologies) was added and the cells were incubated at 37 °C for 30 min and then monitored (ex, 535/em, 617 nm) using the BioTek Synergy H1 plate reader [33]. Fluorescent images were obtained using a fluorescence microscope (Nikon Eclipse 80i, Nikon, Tokyo, Japan) using red filters to visualize the PI-stained cells. This microscope was equipped with the Nikon DS-Ri2 high-definition color digital camera and the Nikon software NIS-elements F imaging software for subsequent analyses [34].

### Membrane potential assays

We monitored the effects of AA exposure by fluorescence assay using the carbocyanine dye, 3,3'-diethylthiacarbocyanine iodide [DisC2(3)] (Invitrogen), which is a good indicator of membrane potential. A graded series of AA concentrations was prepared (1 × , 1/2 × , 1/4 × , 1/8 × , 1/16 × MIC). HA corresponding to the pH of each concentration of AA and colistin (2 μg/mL) served as the positive control was added to mid-log-phase cells. The cultures were sampled at 2 h. The membrane potential was measured using the potentiometric fluorescent probe [DisC2(3)]. Mid-log-phase cells were diluted to an OD_600_ of 0.4. The culture was incubated at 37℃ for 15 min after [DisC2(3)] was added to a final concentration of 2 mM. The plates were monitored (ex, 488 nm; em, 620 nm) on the BioTek Synergy H1 plate reader [9].

### Quantitative reverse transcription PCR (qRT-PCR)

The effects of AA on the expression levels of *P. aeruginosa* QS circuit genes (i.e., *lasR*, *rhlR*, *rhlI*, *rhlA*, and *pqsA*) and T3SS circuit genes (i.e., *exoT*, *exoS*, *exsA*, and *exoY*) and the flagellin gene (*fliC*) were evaluated by qRT-PCR, as described previously [35–38]. *P. aeruginosa* TL1671 and TL2314 was incubated in fresh LB broth at 37℃ under 180 rpm until reaching the logarithmic growth phase (OD_600_ 0.5–0.6). The cultures were then treated with 1/2 × MIC of AA or no AA (control) for 4 h. Total RNA was extracted from planktonic bacteria using the RNeasy Mini Kit (Qiagen, Valencia, CA, USA) in accordance with the manufacturer’s instructions. Purified RNA was reverse transcribed into cDNA using the Cdna Synthesis Kit (TaKaRa, Tokyo, Japan) in accordance with the manufacturer’s instructions. The gene expression levels were measured with qRT-PCR using the TB Green Premix Ex Taq II (Tli RNase H Plus) (2 ×) (Takara) with specific primers listed in Table S2. We use the *rpsL* gene as the reference gene. In the analysis of qRT-PCR results, according to the reference [36, 39], normalized expression of each gene was calibrated against corresponding mRNA expression by control group (no AA treated group). Control group as an internal standard was assigned a value of 1. 2^−ΔΔCt^ was used to calculate the multiples of gene expression in the 1/2MIC group relative to the control group.

### Statistical analysis

The statistical significance of the differences between the control and experimental groups was evaluated by the Student’s *t*-test. For all analyses: *P < 0.05, **P < 0.01, ***P < 0.001, ****P < 0.0001, ns *P* > 0.05.

## Results

### AA has antibacterial and antibiofilm effects on COL-R *P. aeruginosa*

**Fig. 1:**
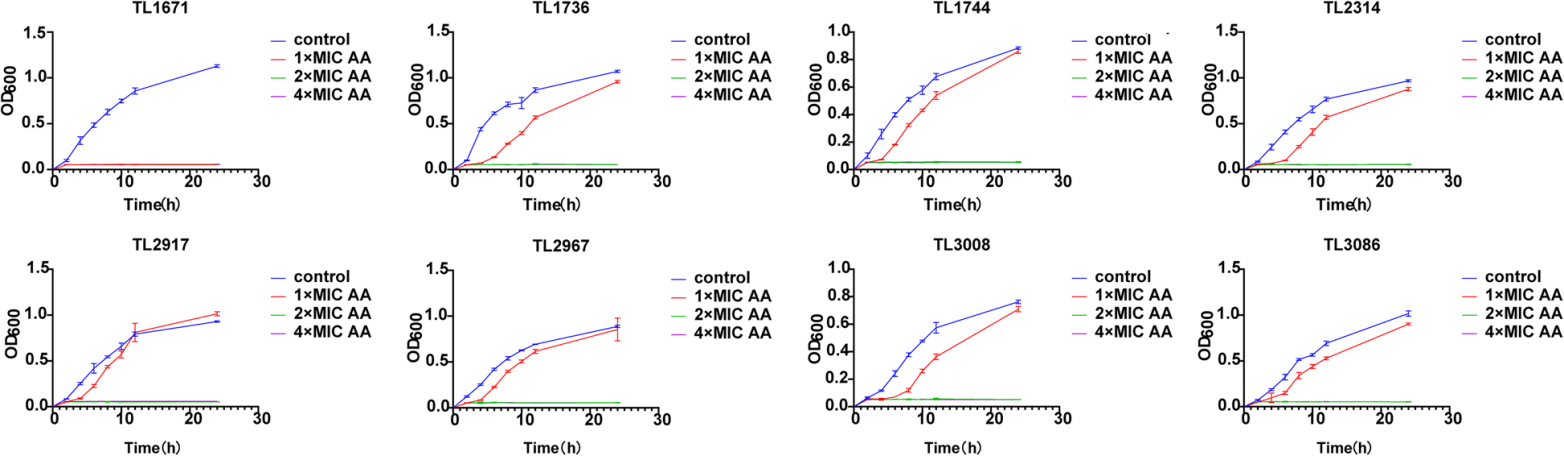
Growth curves of different concentrations of AA (4 × MIC, 2 × MIC, 1 × MIC) and no AA (control) against 8 COL-R *P. aeruginosa.* The experiments were performed thrice. The data were expressed as the mean ± standard deviation

Table S1 in the supplemental material shows the MIC of 8 COL-R *P. aeruginosa*. The results indicated that these eight strains had PmrB and PhoQ substitutions, and MIC of 8 *P. aeruginosa* to colistin was higher than 4 μg/ mL, suggesting that they were all resistant to colistin. However, the MIC of these 8 COL-R *P. aeruginosa* strains to AA was 0.039%-0.078%. Furthermore, growth curves revealed that 0.078% AA could inhibit the growth of these 8 COL-R *P. aeruginosa* strains within 24 h, with no rebound phenomenon occurring (Fig. 1).

**Fig. 2:**
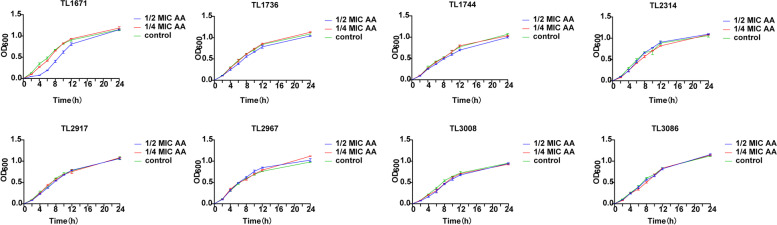
Growth curves of 8 COL-R *P. aeruginosa* treated with AA at the subinhibitory concentrations (1/2 × MIC, 1/4 × MIC) and no AA (control). The experiments were performed thrice. The data are expressed as the mean ± standard deviation

**Fig. 3:**
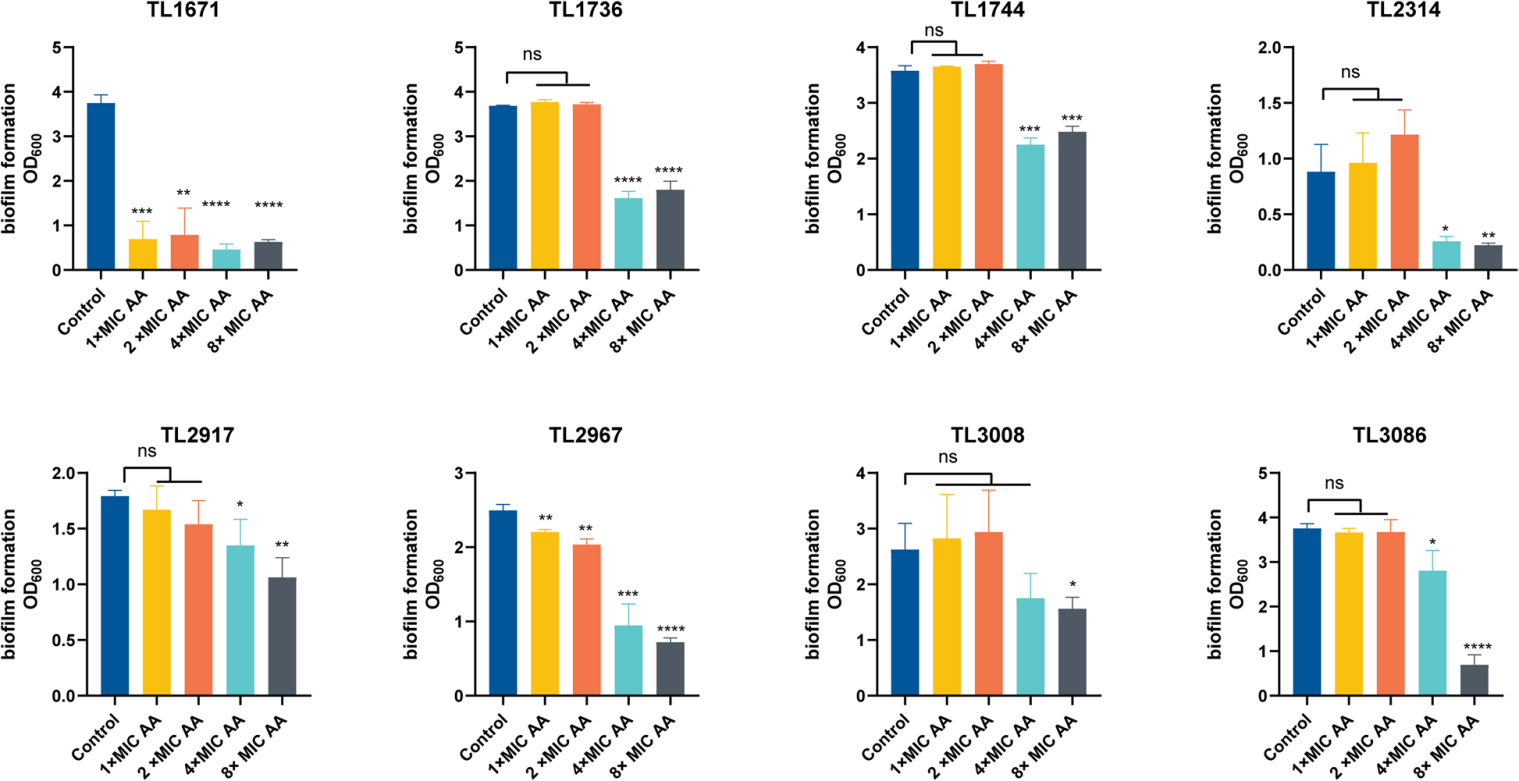
Biofilm eradication effects of AA at different concentrations (8 × MIC, 4 × MIC, 2 × MIC, 1 × MIC) and no AA (control) on 8 COL-R *P. aeruginosa*. Data were analyzed by Student’s *t-*test; ns, not statistically significant; *P < 0.05; **P < 0.01; ***P < 0.001;****P < 0.0001. The experiments were performed thrice. The data are expressed as the mean ± standard deviation

Figure 2 shows that experimental subinhibitory concentrations of AA did not affect the growth of these 8 COL-R *P. aeruginosa* strains. Subsequent detection of the influence of subinhibitory concentrations of AA on biofilm-formation suggested that the subinhibitory concentrations of AA could not inhibit the formation of COL-R *P. aeruginosa* biofilm (Figure S1). However, 4 × MIC and 8 × MIC AA had a good effect on the eradication of biofilms in most strains (Fig. 3).

### AA can reduce the virulence of *P. aeruginosa* in subinhibitory concentrations

**Fig. 4:**
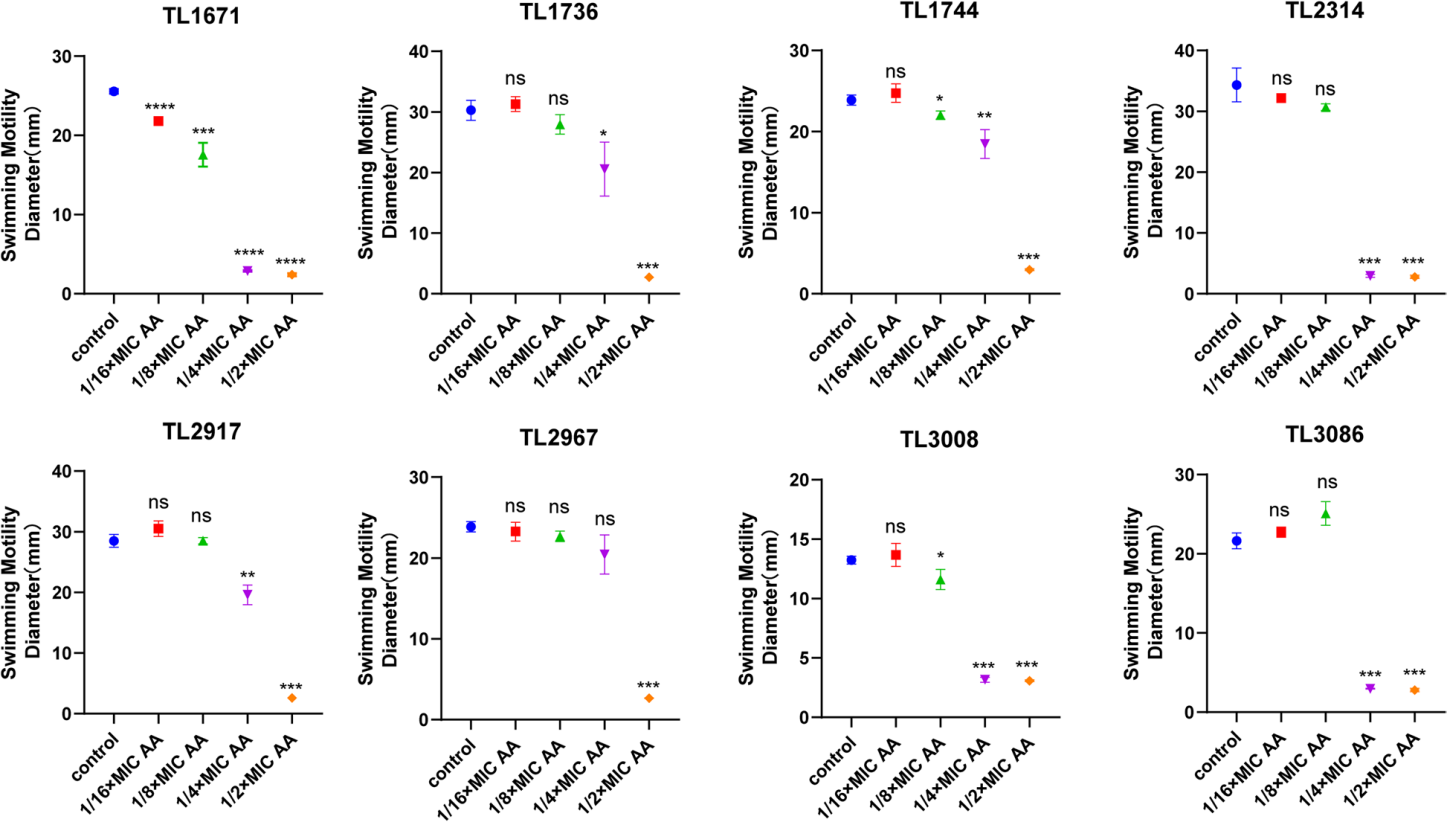
The effect of AA at subinhibitory concentrations (1/2 × MIC, 1/4 × MIC, 1/8 × MIC, 1/16 × MIC) and no AA (control) on the swimming activity against 8 COL-R *P. aeruginosa*. Data were analyzed by Student’s *t*-test; ns, not statistically significant; *P < 0.05; **P < 0.01; ***P < 0.001; ****P < 0.0001. The experiments were performed thrice. The data were expressed as the mean ± standard deviation

**Fig. 5:**
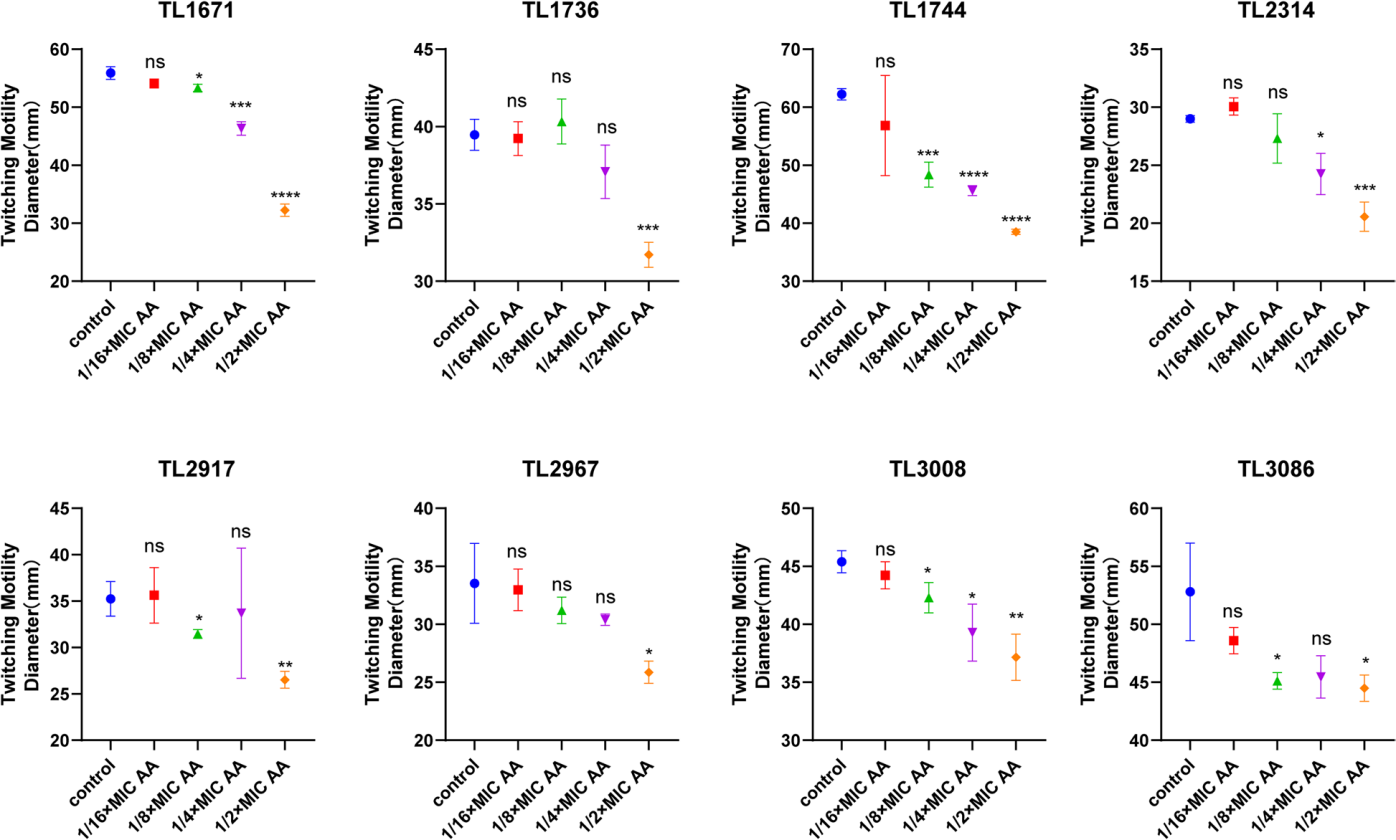
The effect of AA at subinhibitory concentrations (1/2 × , 1/4 × , 1/8 × , 1/16 × MIC) and no AA (control) on the twitching activity against 8 COL-R *P. aeruginosa*. Data were analyzed by Student’s *t-*test; ns, not statistically significant; *P < 0.05; **P < 0.01; ***P < 0.001; ****P < 0.0001. The experiments were performed thrice. The data were expressed as the mean ± standard deviation

**Fig. 6:**
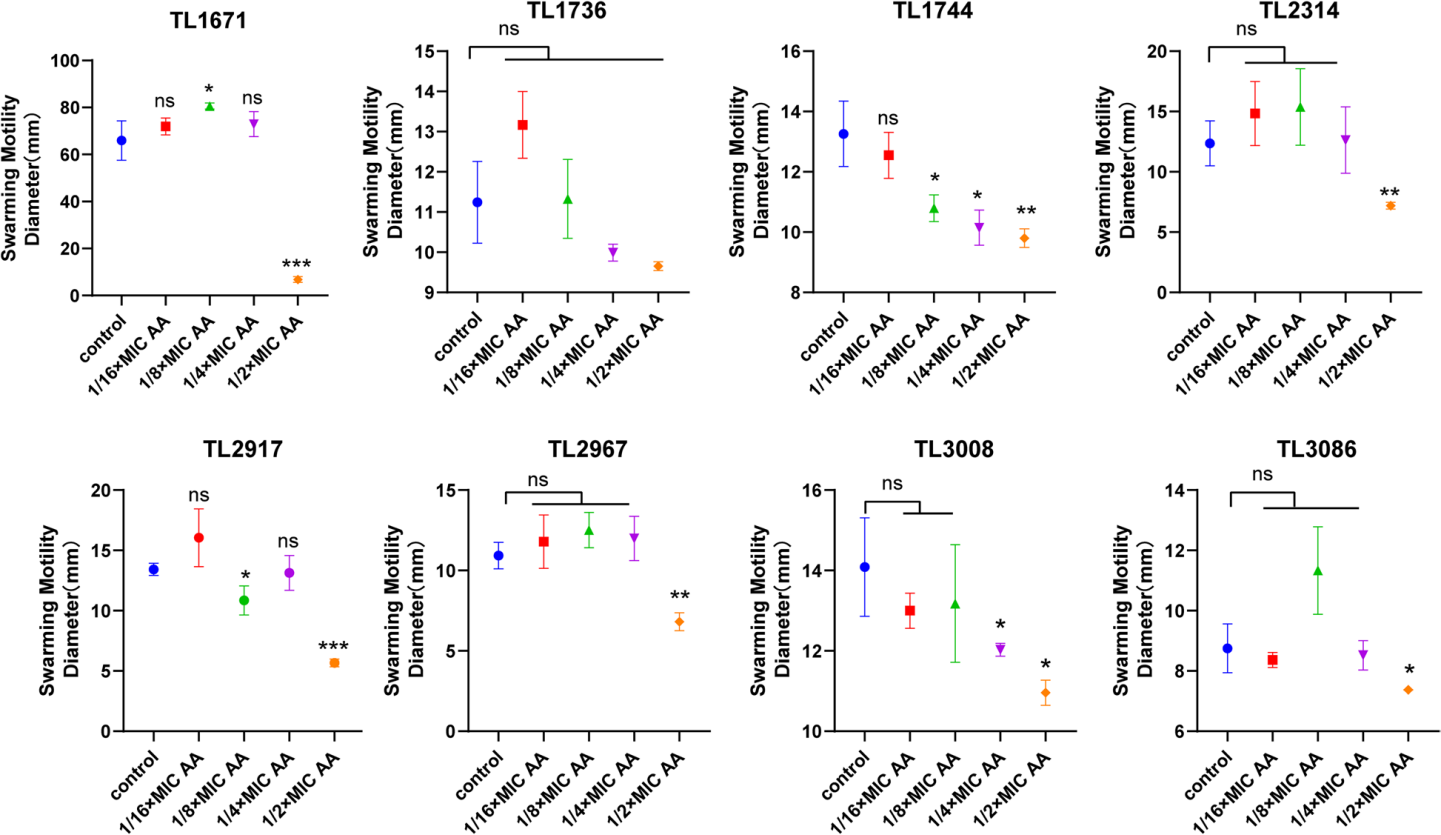
The effect of AA at subinhibitory concentrations (1/2 × , 1/4 × , 1/8 × , 1/16 × MIC) and no AA (control) on the swarming activity against 8 COL-R *P. aeruginos*a. Data were analyzed by Student’s *t*-test; ns, not statistically significant; *P < 0.05; **P < 0.01; ***P < 0.001; ****P < 0.0001. The experiments were performed thrice. The data were expressed as the mean ± standard deviation

Most COL-R *P. aeruginosa* motility assays revealed that 1/2MIC and 1/4MIC AA could inhibit the swimming and twitching motility of most COL-R *P. aeruginosa* (Fig. 4, Fig. 5, Figure S2). Except for TL1736, 1/2MIC AA could suppress the swarming motility of the other strains, indicating that AA at subinhibitory concentrations could inhibit the flagellar movement and type IV pili movement of COL-R *P. aeruginosa* (Fig. 6, Figure S2B). Meanwhile, we detected the effects of different concentrations of AA and the corresponding proton gradient on motility of *P. aeruginosa* TL2314. The results revealed that the proton gradient did not influence the swimming, twitching and swarming motility of *P. aeruginosa* (Figure S2).

**Fig. 7:**
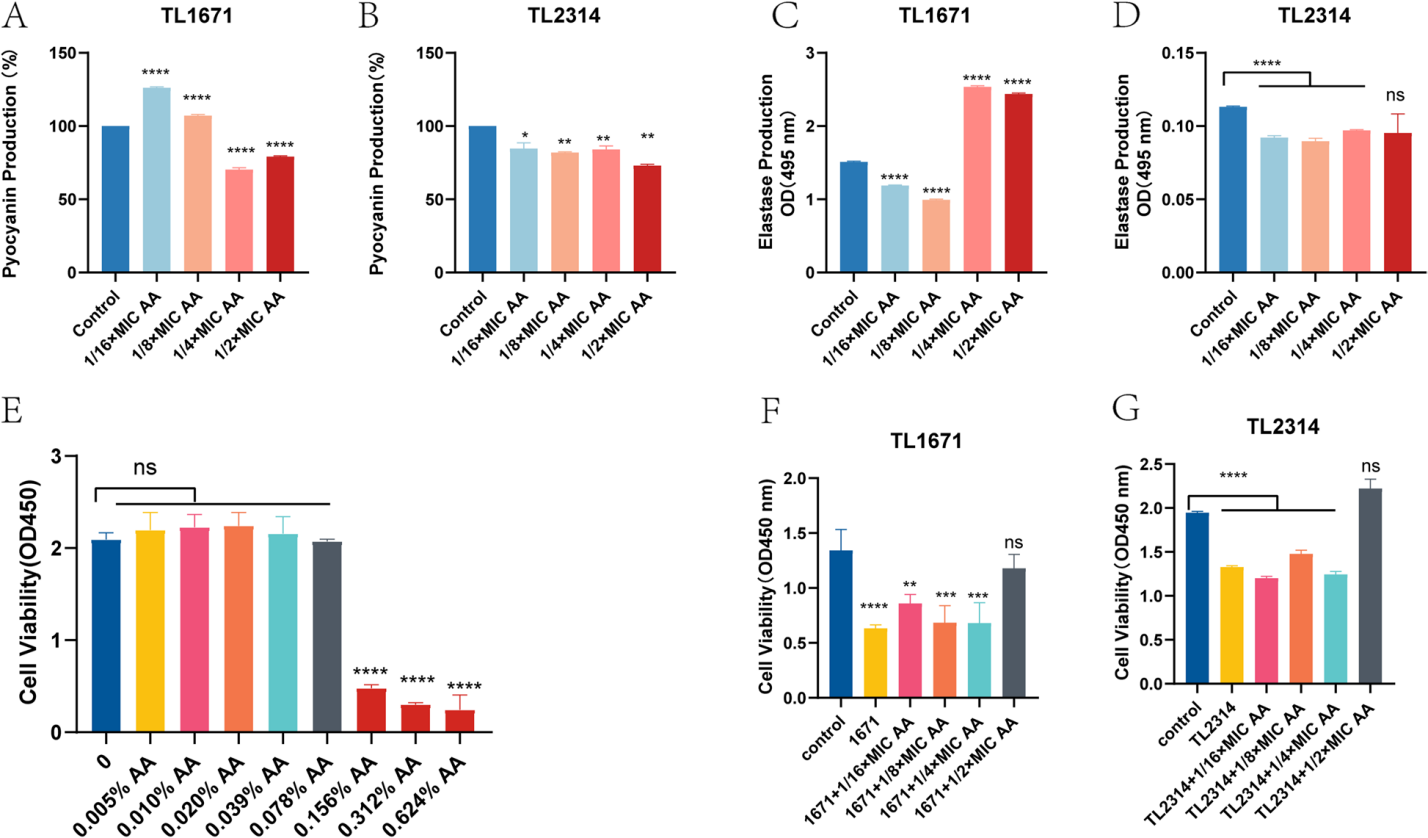
﻿The effect of AA on the virulence of COL-R *P. aeruginosa*. A, B The pyocyanin production of TL1671 and TL2314 after being treated with a graded series of AA concentrations and no AA (control); C, D The elastase production of TL1671 and TL2314 after treatment with a graded series of AA concentrations and no AA (control); E Cytotoxicity of AA to RAW264.7; F, G Cytotoxicity of AA-treated TL1671 and TL2314 bacterial supernatant to RAW264.7. Data were analyzed by Student’s *t*-test; ns, not statistically significant; *P < 0.05; **P < 0.01; ***P < 0.001; ****P < 0.0001. The experiments were performed thrice. The data were expressed as the mean ± standard deviation

To detect the influence of AA on *P. aeruginosa* virulence factors, two strains, TL1671 and TL2314 were randomly selected to detect the synthesis of elastase and pyocyanin in the presence of subinhibitory concentrations of AA. Figure 7A–D indicates that AA can inhibit the production of pyocyanin and elastase. Furthermore, cell viability assays showed that 0.078% AA was not cytotoxic (Fig. 7E). Compared to the untreated bacterial supernatant, the bacterial supernatant treated with 1/2 × MIC AA had limited pathogenicity to macrophages (Fig. 7F, G). Finally, subinhibitory concentrations of AA could inhibit the virulence of COL-R *P. aeruginosa.*

### The potential mechanism of AA

**Fig. 8:**
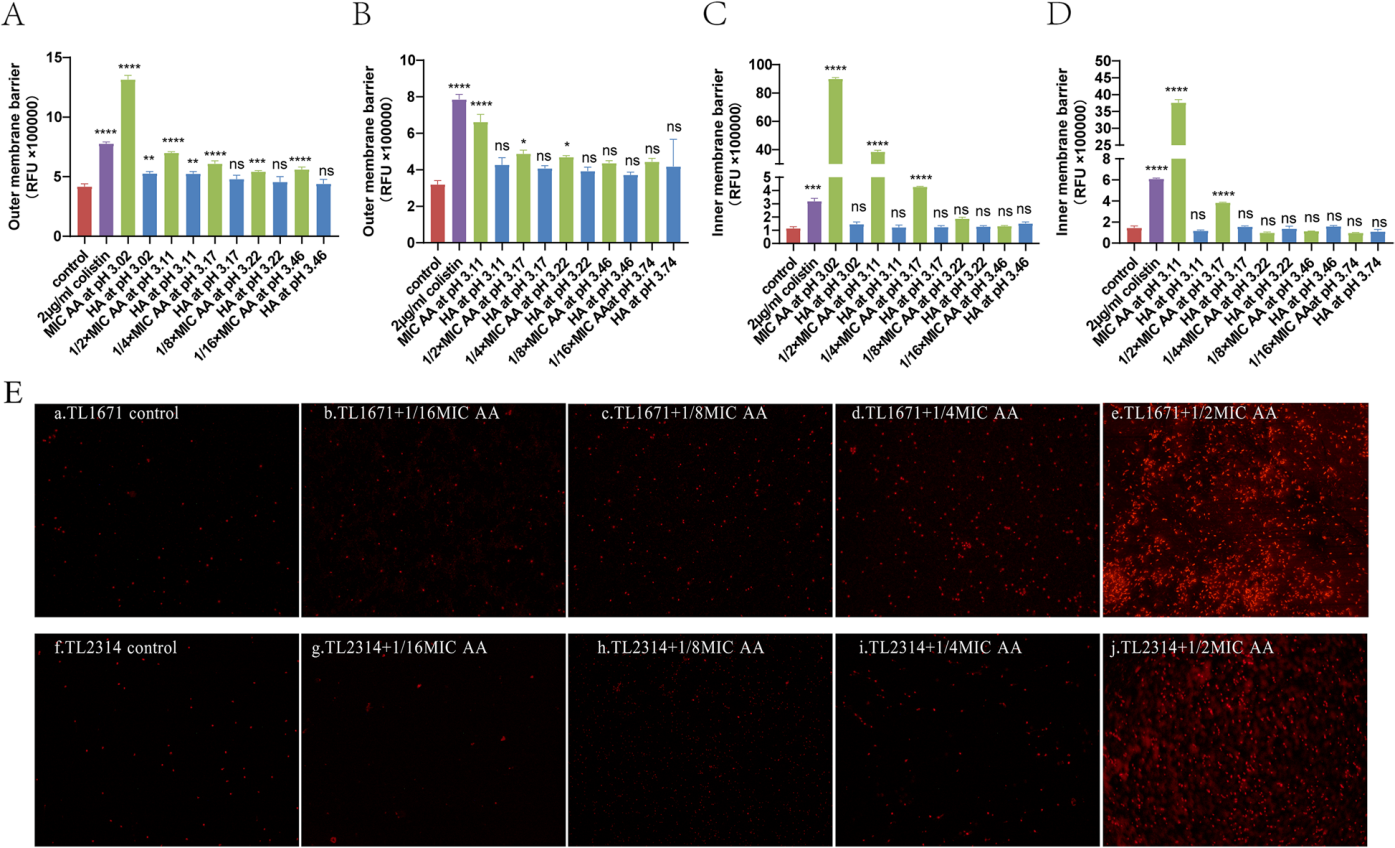
The potential antibacterial mechanism of AA. **A**, **B** Effects of AA and HA corresponding to the pH of each concentration of AA on the outer membrane permeability of TL1671 and TL2314, respectively; **C**, **D** Effects of AA and HA corresponding to the pH of each concentration of AA on the inner membrane permeability of TL1671 and TL2314 by using a plate reader, respectively; **E** Effect of AA on the inner membrane permeability of TL1671 and TL2314 by a fluorescence microscope. a TL1671 treated with the LB broth control; b–e TL1671 treated with 1/16 × MIC, 1/8 × MIC, 1/4 × MIC, 1/2 × MIC AA, respectively; f TL2314 treated with the LB broth control; g–j TL1671 exposed to 1/16 × MIC, 1/8 × MIC, 1/4 × MIC, 1/2 × MIC AA, respectively. Data were analyzed by Student’s *t*-test; ns, not statistically significant; *P < 0.05; **P < 0.01; ***P < 0.001; ****P < 0.0001. The experiments were performed thrice. The data were expressed as the mean ± standard deviation

**Fig. 9:**
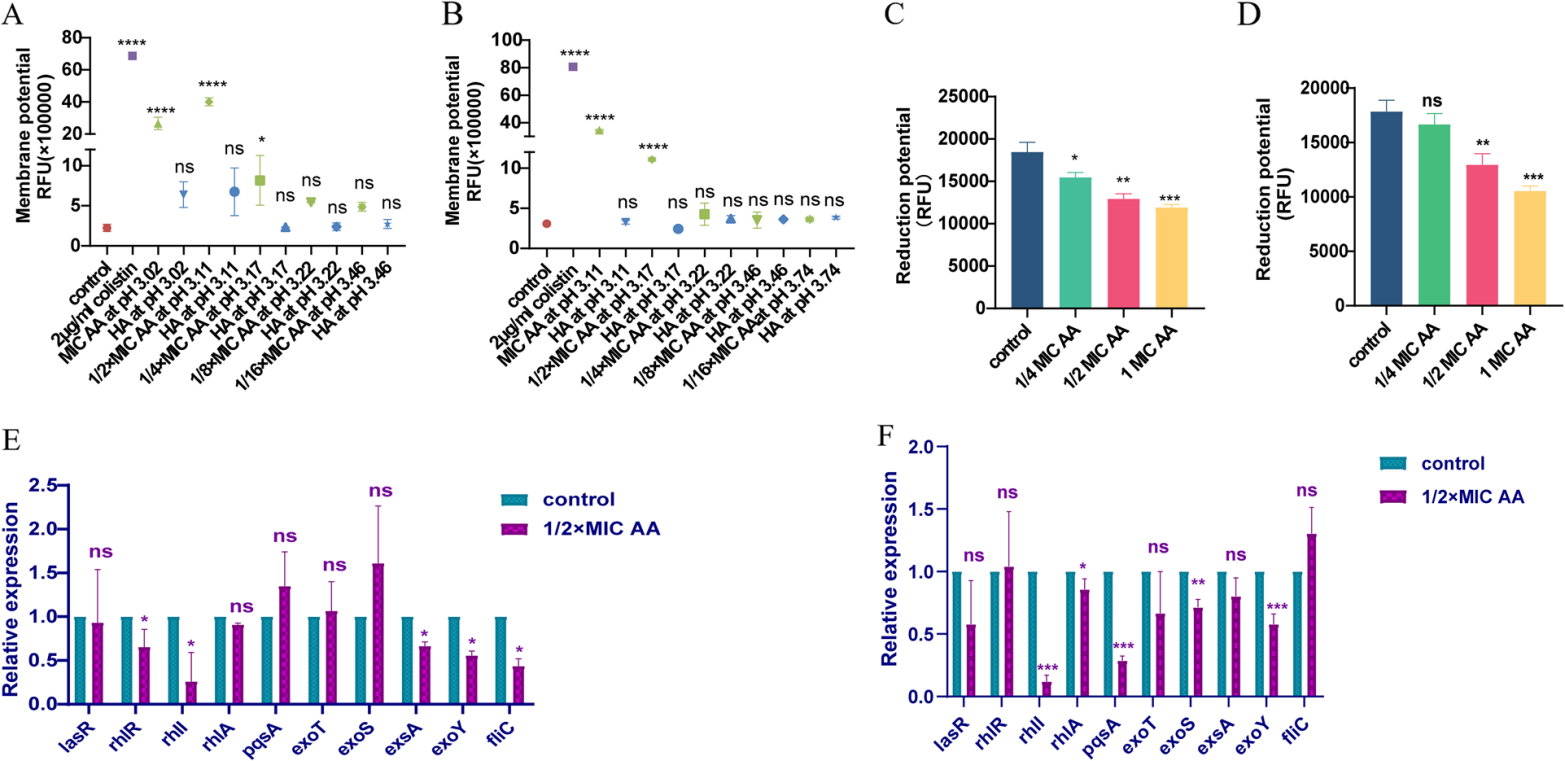
The potential antibacterial mechanism of AA. **A**, **B** The effect of AA and HA corresponding to the pH of each concentration of AA on the membrane potential of TL1671 and TL2314; **C**, **D** The effect of AA on the respiratory rate of TL1671 and TL2314; **E, F** The effect of AA on QS-related and T3SS-related gene expression in *P. aeruginosa*. A ‘no AA’ group served as the control. **E** TL1671 (no AA) and TL1671 + 1/2 × MIC AA; **F** TL2314 (no AA) and TL2314 + 1/2 × MIC AA. The data were expressed as the means ± SD (*n* = 3). Data were analyzed by Student’s *t*-test; ns, not statistically significant; *P < 0.05; **P < 0.01; ***P < 0.001; ****P < 0.0001. The experiments were performed thrice. The data were expressed as the mean ± standard deviation

AA has good antibacterial and antibiofilm activities against COL-R *P. aeruginosa.* What is the potential mechanism? AA was tested for its effect on the outer and inner membrane permeability of *P. aeruginosa* using NPN and PI assays, and the results of NPN assays showed that AA could increase the outer membrane permeability of TL1671 and TL2314 compared to the control group (Fig. 8A, B). Moreover, this effect may be related to pH to some extent and does not depend entirely on pH influence because HA with the same pH as MIC and 1/2MIC AA can promote the increase of TL1671 outer membrane permeability, it is still different from AA treatment. In addition, we tried to add sodium acetate solution with the same concentration as AA as the control group, and the effect of carboxylate ions on membrane permeability of *P. aeruginosa* was detected. The results showed that sodium acetate corresponding to 1 × MIC and 1/2 × MIC AA did not affect the permeability of the outer membrane and inner membrane of *P. aeruginosa* (Figure S3). The results of PI assays performed with a plate reader and a fluorescence microscope revealed that AA could increase the inner membrane permeability of TL1671 and TL2314 compared to the control group (Fig. 8C, D, E), which was not pH dependent. Additionally, AA may polarize the membrane potential, independent of pH (Fig. 9A, B). Furthermore, we used the indicator resazurin to monitor respiration in COL-R *P. aeruginosa* after AA treatment, and the results showed that AA inhibits *P. aeruginosa* reduction potential in a concentration-dependent manner (Fig. 9C, D).

AA can impede *P. aeruginosa* motility and the formation of pyocyanin and elastase. The type III secretion system (T3SS) and quorum sensing (QS) system can regulate *P. aeruginosa* motility and virulence factor secretion [40]. Therefore, we evaluated the expression of QS-regulated genes, T3SS circuit genes, and the flagellin gene in *P. aeruginosa* in response to AA. Ct values were used to calculate the relative expression of genes in *P. aeruginosa* TL1671 and TL2314. The results demonstrated that AA inhibits the relative expression of some T3SS-related and QS-related genes, as shown in Fig. 9E and F, suggesting that AA may inhibit the virulence of COL-R *P. aeruginosa* by inhibiting the expression of T3SS and QS genes.

## Discussion

*P. aeruginosa* is a leading source of acute hospital infections and pneumonia, with high morbidity and mortality, including healthcare-associated pneumonia and chronic obstructive pulmonary disease (COPD) [41]. With the widespread use of colistin, the emergence of COL-R *P. aeruginosa* is increasing, posing significant clinical anti-infection prevention and treatment issues. AA, the active component of vinegar, has been reported to cure *P. aeruginosa* wound infections [42] and be an effective tuberculocidal disinfectant [41]. Although the disinfectant properties of organic acids, AA, are widely recognized, they have rarely been mentioned in bactericides evaluations [41]. Furthermore, it is unknown if AA has good antibacterial, antibiofilm, and anti-virulence activities against COL-R *P. aeruginosa* derived from sputum and wound, as well as the potential antibacterial mechanism of AA.

The antibacterial, antibiofilm, and anti-virulence properties of AA against COL-R *P. aeruginosa,* as well as the potential mechanism, were investigated in this study. Compared to the concentrations used in Madhusudhan VL’s research, the MIC of AA to *P. aeruginosa* was substantially lower than the 1% (v/v) concentration commonly used in the clinic [43]. The antimicrobial susceptibility and growth curve results showed that 0.078% (v/v) AA had good antibacterial activity against COL-R *P. aeruginosa*, consistent with Sloss's study [9]. Unlike Halstead’s work, which revealed that subinhibitory concentrations of AA could inhibit biofilm formation [44], we found that subinhibitory concentrations of AA did not inhibit biofilm formation but 4 × MIC and 8 × MIC AA could eradicate biofilms in most strains. These results indicate that AA has good antibacterial and antibiofilm activities against COL-R *P. aeruginosa*.

Motility is required for the initial attachment of bacterial cells to biotic and abiotic surfaces, thereby contributing to MDR among pathogens. For the reasons mentioned above, *P. aeruginosa* near-surface movements (e.g., swimming, swarming, and twitching) have been regarded as a virulence property [45]. The results of motility assays showed that AA inhibited COL-R *P. aeruginosa* swimming, swarming, and twitching. Similarly, we tested the impact of proton gradient on *P. aeruginosa* motility using HA at the same pH as AA as a control. Surprisingly, we discovered that *P. aeruginosa* motility did not appear to be inhibited by a weak acidic pH. It was reported that lactic acid can also inhibit the motility of *P. aeruginosa* and the expression of QS related genes of *P. aeruginosa* [46, 47], and hibiscus acid can inhibit the flagellum movement of *Salmonella enterica*. In line with the findings of our study, the weak acidic pH is not the reason that hibiscus acid affects the movement of *Salmonella enterica* [48]. Elastase and pyocyanin are other *P. aeruginosa* virulence factors that aid colonization and bacterial immune evasion. In vitro, suppression of bacterial proteases such as *P. aeruginosa* elastase resulted in bacterial biofilm disintegration. Pyocyanin also alters the host immune response in several ways to facilitate immune system evasion and the establishment of a chronic infection. Subinhibitory doses of AA were shown to suppress the synthesis of pyocyanin and elastase in pyocyanin and elastase assays. In comparison to the untreated bacterial supernatant, the results showed that the cytotoxicity of the TL1671 and TL2314 supernatants treated with 1/2 × MIC AA was also non-existent. Therefore, AA not only exhibited strong antibacterial and antibiofilm action but also inhibited *P. aeruginosa* virulence*.*

AA is a weak organic acid capable of ionizing hydrogen and acetate ions. Low pH has been shown in studies to restrict bacterial growth, transcription, and translation [45]. However, several studies have found that the bactericidal action of AA is not related to the decrease in pH; rather, the AA molecule in its non-dissociated form kills bacteria since HA of the same pH cannot exert antibacterial activity [49]. What are the probable repercussions for the different cell components in the presence of AA? We explored the effect of AA on the cell membrane permeability of *P. aeruginosa* and found that AA could promote the permeability of the outer membrane of bacteria, but the influence of AA on the permeability of the outer membrane of *P. aeruginosa* was not entirely contributed by pH, which indicated that fully dissociated acid can partly cause an increase in the permeability of membranes. The additional membrane disintegrating effect demonstrated here for AA is likely due to undissociated AA molecules, which was further supported by sodium acetate corresponding to 1 × MIC and 1/2 × MIC AA did not affect the permeability of the outer membrane and inner membrane of *P. aeruginosa.* Our results are consistent with earlier findings on the underlying mechanism of organic acid lactic acid causing sublethal damage to Gram-negative bacteria [50]. However, we need to further explore how the AA molecules in non-dissociated plays its role in the future. Futhermore, AA increased the inner membrane permeability of *P. aeruginosa,* which didn’t rely on pH*,* implying that the AA molecule may damage the outer and inner membrane barrier of *P. aeruginosa*. Then, following AA treatment, we discovered *P. aeruginosa* impact on cell membrane potential indepent of pH. In addition, does AA damage *P. aeruginosa* enzymes? AA was discovered to inhibit *P. aeruginosa* reduction potential. *P. aeruginosa* may increase the expression of respiratory chain complexes that pump protons out of the cell while decreasing the expression of the ATP synthase, which pumps protons into the cell during ATP synthesis [45].

QS is a global regulatory mechanism that enables bacteria to communicate with each other by producing autoinducers (AI) molecules in the population [51]. The T3SS system comprises the type III secretion and translocation machinery, regulators, effectors, and effector-specific chaperones, which permit direct delivery of several bacterial effector proteins into eukaryotic host cells [52]. *P. aeruginosa* pathogenicity is dependent on motility and the production of virulence factors such as pyocyanin and elastase, which is controlled by QS and T3SS systems. The effect of AA on the expression of QS-related and T3SS-related genes was investigated preliminarily in this study. The qRT-PCR results showed that 1/2 × MIC AA could inhibit some QS-related and T3SS-related genes, but how AA regulates *P. aeruginosa* virulence via QS and T3SS systems remains unknown.

In conclusion, AA possesses good antibacterial, antibiofilm, and anti-virulence activities. According to our findings, a high concentration of AA (> 0.156% v/v) can be used to sterilize biofilm-prone surgical instruments, hospital disinfection, and wound external use. A low concentration of AA (0.00975–0.039% v/v) was used as an anti-virulence agent for adjuvant treatment of COL-R *P. aeruginosa*.

## Supplementary Information

**Additional file 1: Figure S1. **Biofilm-formation inhibition of AA at different concentrations (1/2× MIC, 1/4×MIC, 1/8×MIC, 1/16×MIC) and no AA (control) on 8 COL-R *P. aeruginosa*. Data were analyzed by Student’st-test; ns, not statistically significant; *P < 0.05; **P < 0.01; ***P < 0.001;****P < 0.0001. The experiments were performed thrice. The data are expressed as the mean ±stand. **Figure S2.** Effect of AA at subinhibitory concentrations (1/2×, 1/4×, 1/8×, 1/16× MIC) and no AA (control) on the motility against *P. aeruginosa* TL2314. **Figure S3. **The potential antibacterial mechanism of AA. **Table S1. **The antimicrobial susceptibility of colistin and AA against COL-R *P. aeruginos*a. **Table S2. **Primers used for qRT-PCR. 

## Data Availability

All data generated or analysed during this study are included in this published article and its supplementary information files. The datasets used and analysed during the current study available from the corresponding author on reasonable request.

## Abbreviations

*P. aeruginosa*: *Pseudomonas aeruginosa*
COL-R: Colistin-resistant
MIC: Minimum inhibitory concentration
CF: Cystic fibrosis
WHO: World health organisation
MDR: Multi-drug resistant
XDR: Extensively drug-resistant
MALDI-TOF/MS: Matrix-assisted laser desorption/ionization time-of-flight mass spectrometry
CAMHB: Cationic adjusted Mueller–Hinton broth
LB: Luria Broth
PI: Propidium iodide
COPD: Chronic obstructive pulmonary disease

## References

[1] Tashiro Y, Yawata Y, Toyofuku M, Uchiyama H, Nomura N (2013). Interspecies interaction between Pseudomonas aeruginosa and other microorganisms. Microbes Environ.

[2] Watters C, DeLeon K, Trivedi U, Griswold JA, Lyte M, Hampel KJ (2013). Pseudomonas aeruginosa biofilms perturb wound resolution and antibiotic tolerance in diabetic mice. Med Microbiol Immunol.

[3] Meskini M, Esmaeili D (2018). The study of formulated Zoush ointment against wound infection and gene expression of virulence factors Pseudomonas aeruginosa. BMC Complement Altern Med.

[4] Azuama OC, Ortiz S, Quiros-Guerrero L, Bouffartigues E, Tortuel D, Maillot O, et al. Tackling Pseudomonas aeruginosa Virulence by Mulinane-Like Diterpenoids from Azorella atacamensis. Biomolecules. 2020;10(12). 10.3390/biom10121626. 10.3390/biom10121626 PMC7761567 33276611

[5] Vanderwoude J, Fleming D, Azimi S, Trivedi U, Rumbaugh KP, Diggle SP (1937). The evolution of virulence in Pseudomonas aeruginosa during chronic wound infection. Proc Biol Sci.

[6] Jurado-Martin I, Sainz-Mejias M, McClean S. Pseudomonas aeruginosa: An Audacious Pathogen with an Adaptable Arsenal of Virulence Factors. Int J Mol Sci. 2021;22(6). 10.3390/ijms22063128. 10.3390/ijms22063128 PMC8003266 33803907

[7] Abd El-Baky RM, Masoud SM, Mohamed DS, Waly NG, Shafik EA, Mohareb DA (2020). Prevalence and Some Possible Mechanisms of Colistin Resistance Among Multidrug-Resistant and Extensively Drug-Resistant Pseudomonas aeruginosa. Infect Drug Resist.

[8] Schaefer P, Baugh RF (2012). Acute otitis externa: an update. Am Fam Physician.

[9] Sloss JM, Cumberland N, Milner SM (1993). Acetic acid used for the elimination of Pseudomonas aeruginosa from burn and soft tissue wounds. J R Army Med Corps.

[10] Currence WW (1952). Acetic acid aerosol in treatment of purulent bronchiectasis due to Pseudomonas aeruginosa. AMA Am J Dis Child.

[11] Kjeldsen M, Homoe P, Kirstine Nielsen A, Crone S, Norskov Kragh K, Bjarnsholt T (2020). Eradication of biofilms on tympanostomy tubes with acetic acid treatment: an in vitro study. APMIS.

[12] Bjarnsholt T, Alhede M, Jensen PO, Nielsen AK, Johansen HK, Homoe P (2015). Antibiofilm Properties of Acetic Acid. Adv Wound Care (New Rochelle).

[13] Madhusudhan VL (2016). Efficacy of 1% acetic acid in the treatment of chronic wounds infected with Pseudomonas aeruginosa: prospective randomised controlled clinical trial. Int Wound J.

[14] Nagoba BS, Deshmukh SR, Wadher BJ, Patil SB (1997). Acetic acid treatment of pseudomonal postoperative wound infection. J Hosp Infect.

[15] Ilic BS, Kocic BD, Ciric VM, Cvetkovic OG, Miladinovic DL (2014). An in vitro synergistic interaction of combinations of Thymus glabrescens essential oil and its main constituents with chloramphenicol. ScientificWorldJournal.

[16] Dortet L, Broda A, Bernabeu S, Glupczynski Y, Bogaerts P, Bonnin R (2020). Optimization of the MALDIxin test for the rapid identification of colistin resistance in Klebsiella pneumoniae using MALDI-TOF MS. J Antimicrob Chemother.

[17] Prateeksha, Rao CV, Das AK, Barik SK, Singh BN. ZnO/Curcumin Nanocomposites for Enhanced Inhibition of Pseudomonas aeruginosa Virulence via LasR-RhlR Quorum Sensing Systems. Mol Pharm. 2019;16(8):3399–413. 10.1021/acs.molpharmaceut.9b00179. 10.1021/acs.molpharmaceut.9b00179 31260316

[18] Fila G, Krychowiak M, Rychlowski M, Bielawski KP, Grinholc M (2018). Antimicrobial blue light photoinactivation of Pseudomonas aeruginosa: Quorum sensing signaling molecules, biofilm formation and pathogenicity. J Biophotonics.

[19] Mok N, Chan SY, Liu SY, Chua SL (2020). Vanillin inhibits PqsR-mediated virulence in Pseudomonas aeruginosa. Food Funct.

[20] Baldelli V, D'Angelo F, Pavoncello V, Fiscarelli EV, Visca P, Rampioni G (2020). Identification of FDA-approved antivirulence drugs targeting the Pseudomonas aeruginosa quorum sensing effector protein PqsE. Virulence.

[21] Kohler T, Curty LK, Barja F, van Delden C, Pechere JC (2000). Swarming of Pseudomonas aeruginosa is dependent on cell-to-cell signaling and requires flagella and pili. J Bacteriol.

[22] Luo J, Dong B, Wang K, Cai S, Liu T, Cheng X (2017). Baicalin inhibits biofilm formation, attenuates the quorum sensing-controlled virulence and enhances Pseudomonas aeruginosa clearance in a mouse peritoneal implant infection model. PLoS ONE.

[23] Lau GW, Hassett DJ, Ran H, Kong F (2004). The role of pyocyanin in Pseudomonas aeruginosa infection. Trends Mol Med.

[24] Skindersoe ME, Alhede M, Phipps R, Yang L, Jensen PO, Rasmussen TB (2008). Effects of antibiotics on quorum sensing in Pseudomonas aeruginosa. Antimicrob Agents Chemother.

[25] Dong L, Sun L, Hu X, Nie T, Pang J, Wang X (2021). Ostarine attenuates pyocyanin in Pseudomonas aeruginosa by interfering with quorum sensing systems. J Antibiot (Tokyo).

[26] Everett MJ, Davies DT (2021). Pseudomonas aeruginosa elastase (LasB) as a therapeutic target. Drug Discov Today.

[27] Wang H, Chu W, Ye C, Gaeta B, Tao H, Wang M (2019). Chlorogenic acid attenuates virulence factors and pathogenicity of Pseudomonas aeruginosa by regulating quorum sensing. Appl Microbiol Biotechnol.

[28] Dombach JL, Quintana JLJ, Detweiler CS. Staphylococcal Bacterial Persister Cells, Biofilms, and Intracellular Infection Are Disrupted by JD1, a Membrane-Damaging Small Molecule. mBio. 2021;12(5):e0180121. 10.1128/mBio.01801-21. 10.1128/mBio.01801-21 PMC8510524 34634935

[29] Czekanska EM (2011). Assessment of cell proliferation with resazurin-based fluorescent dye. Methods Mol Biol.

[30] Curran CS, Bolig T, Torabi-Parizi P (2018). Mechanisms and Targeted Therapies for Pseudomonas aeruginosa Lung Infection. Am J Respir Crit Care Med.

[31] Wang G, Brunel JM, Preusse M, Mozaheb N, Willger SD, Larrouy-Maumus G (2022). The membrane-active polyaminoisoprenyl compound NV716 re-sensitizes Pseudomonas aeruginosa to antibiotics and reduces bacterial virulence. Commun Biol.

[32] Li F, Xia Q, Ren L, Nie Y, Ren H, Guo X (2022). GSDME Increases Chemotherapeutic Drug Sensitivity by Inducing Pyroptosis in Retinoblastoma Cells. Oxid Med Cell Longev.

[33] Owen CR (1946). Acetic acid inhibition of gram-negative bacilli in culture media. J Bacteriol.

[34] Yao Z, Feng L, Zhao Y, Zhang X, Chen L, Wang L (2022). Thymol Increases Sensitivity of Clinical Col-R Gram-Negative Bacteria to Colistin. Microbiol Spectr.

[35] Chen T, Xu Y, Xu W, Liao W, Xu C, Zhang X (2020). Hypertonic glucose inhibits growth and attenuates virulence factors of multidrug-resistant Pseudomonas aeruginosa. BMC Microbiol.

[36] Zheng X, Zhang X, Zhou B, Liu S, Chen W, Chen L (2022). Clinical characteristics, tolerance mechanisms, and molecular epidemiology of reduced susceptibility to chlorhexidine among Pseudomonas aeruginosa isolated from a teaching hospital in China. Int J Antimicrob Agents.

[37] Zhang Y, Wang L, Chen L, Zhu P, Huang N, Chen T (2022). Novel Insight of Transcription Factor PtrA on Pathogenicity and Carbapenems Resistance in Pseudomonas aeruginosa. Infect Drug Resist.

[38] Abdel-Rhman SH, Rizk DE, Abdelmegeed ES (2020). Effect of Sub-Minimum Inhibitory Concentrations of Tyrosol and EDTA on Quorum Sensing and Virulence of Pseudomonas aeruginosa. Infect Drug Resist.

[39] Quale J, Bratu S, Gupta J, Landman D (2006). Interplay of efflux system, ampC, and oprD expression in carbapenem resistance of Pseudomonas aeruginosa clinical isolates. Antimicrob Agents Chemother.

[40] Munoz-Cazares N, Castillo-Juarez I, Garcia-Contreras R, Castro-Torres VA, Diaz-Guerrero M, Rodriguez-Zavala JS, et al. A Brominated Furanone Inhibits Pseudomonas aeruginosa Quorum Sensing and Type III Secretion, Attenuating Its Virulence in a Murine Cutaneous Abscess Model. Biomedicines. 2022;10(8). 10.3390/biomedicines10081847. 10.3390/biomedicines10081847 PMC9404868 36009394

[41] Cortesia C, Vilcheze C, Bernut A, Contreras W, Gomez K, de Waard J, et al. Acetic Acid, the active component of vinegar, is an effective tuberculocidal disinfectant. mBio. 2014;5(2):e00013–14. 10.1128/mBio.00013-14. 10.1128/mBio.00013-14 PMC3940030 24570366

[42] Rozenblat M, Last O, Fisher S, Ziv M (2019). Acetic acid treatment for toe web infection caused by Pseudomonas Aeruginosa combined with fungal infection: A case series of ten patients. Dermatol Ther.

[43] Khan F, Pham DTN, Oloketuyi SF, Kim YM (2020). Regulation and controlling the motility properties of Pseudomonas aeruginosa. Appl Microbiol Biotechnol.

[44] Oriel JD, Partridge BM, Denny MJ, Coleman JC (1972). Genital yeast infections. Br Med J.

[45] Lund P, Tramonti A, De Biase D (2014). Coping with low pH: molecular strategies in neutralophilic bacteria. FEMS Microbiol Rev.

[46] Kiymaci ME, Altanlar N, Gumustas M, Ozkan SA, Akin A (2018). Quorum sensing signals and related virulence inhibition of Pseudomonas aeruginosa by a potential probiotic strain's organic acid. Microb Pathog.

[47] Azami S, Arefian E, Kashef N (2022). Postbiotics of Lactobacillus casei target virulence and biofilm formation of Pseudomonas aeruginosa by modulating quorum sensing. Arch Microbiol.

[48] Sedillo-Torres IY, Hernandez-Rangel AO, Gomez YGY, Cortes-Avalos D, Garcia-Perez BE, Villalobos-Rocha JC, et al. Hibiscus Acid from Hibiscus sabdariffa L. Inhibits Flagellar Motility and Cell Invasion in Salmonella enterica. Molecules. 2022;27(3). 10.3390/molecules27030655. 10.3390/molecules27030655 PMC8839027 35163919

[49] Maiden MM, Waters CM (2020). Triclosan depletes the membrane potential in Pseudomonas aeruginosa biofilms inhibiting aminoglycoside induced adaptive resistance. PLoS Pathog.

[50] Alakomi HL, Skytta E, Saarela M, Mattila-Sandholm T, Latva-Kala K, Helander IM (2000). Lactic acid permeabilizes gram-negative bacteria by disrupting the outer membrane. Appl Environ Microbiol.

[51] Yang L, Liu Y, Sternberg C, Molin S (2010). Evaluation of enoyl-acyl carrier protein reductase inhibitors as Pseudomonas aeruginosa quorum-quenching reagents. Molecules.

[52] Kong W, Dong M, Yan R, Liang Q, Zhang H, Luo W (2019). A Unique ATPase, ArtR (PA4595), Represses the Type III Secretion System in Pseudomonas aeruginosa. Front Microbiol.

