# Supplementary material for: Evaluation of the antibacterial, antibiofilm, and anti-virulence effects of acetic acid and the related mechanisms on colistin-resistant Pseudomonas aeruginosa
Source: BMC Microbiol. 2022 Dec 19;22:306. doi: 10.1186/s12866-022-02716-6 (PMC9762083; doi:10.1186/s12866-022-02716-6)
Supplement: Supplementary file 1 — Additional file 1: Figure S1. Biofilm-formation inhibition of AA at different concentrations (1/2× MIC, 1/4×MIC, 1/8×MIC, 1/16×MIC) and no AA (control) on 8 COL-R P. aeruginosa. Data were analyzed by Student’st-test; ns, not statistically significant; *P < 0.05; **P < 0.01; ***P < 0.001;****P < 0.0001. The experiments were performed thrice. The data are expressed as the mean ±stand. Figure S2. Effect of AA at subinhibitory concentrations (1/2×, 1/4×, 1/8×, 1/16× MIC) and no AA (control) on the motility against P. aeruginosa TL2314. Figure S3. The potential antibacterial mechanism of AA. Table S1. The antimicrobial susceptibility of colistin and AA against COL-R P. aeruginosa. Table S2. Primers used for qRT-PCR. [file 12866_2022_2716_MOESM1_ESM.pdf]

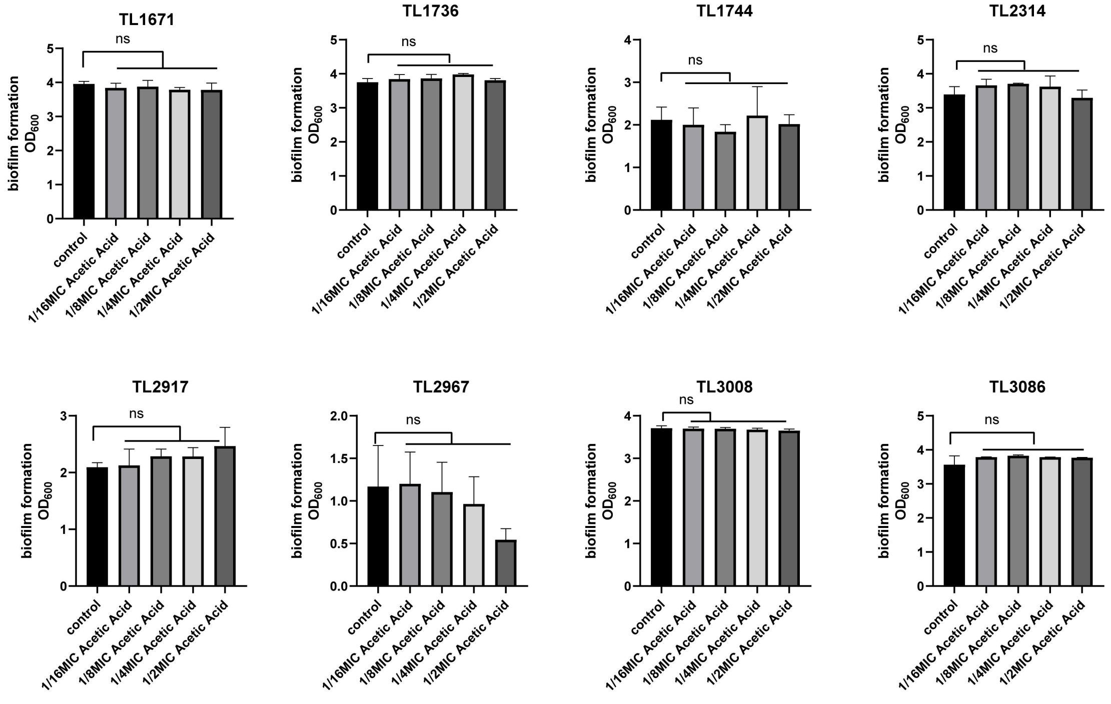

Figure S1 Biofilm-formation inhibition of AA at different concentrations (1/2× MIC, 1/4×MIC, 1/8 ×MIC, 1/16×MIC) and no AA (control) on 8 COL-R *P. aeruginosa*. Data were analyzed by Student's t-test; ns, not statistically significant; *P < 0.05; **P < 0.01; ***P < 0.001;****P < 0.0001. The experiments were performed thrice. The data are expressed as the mean ±standard deviation.

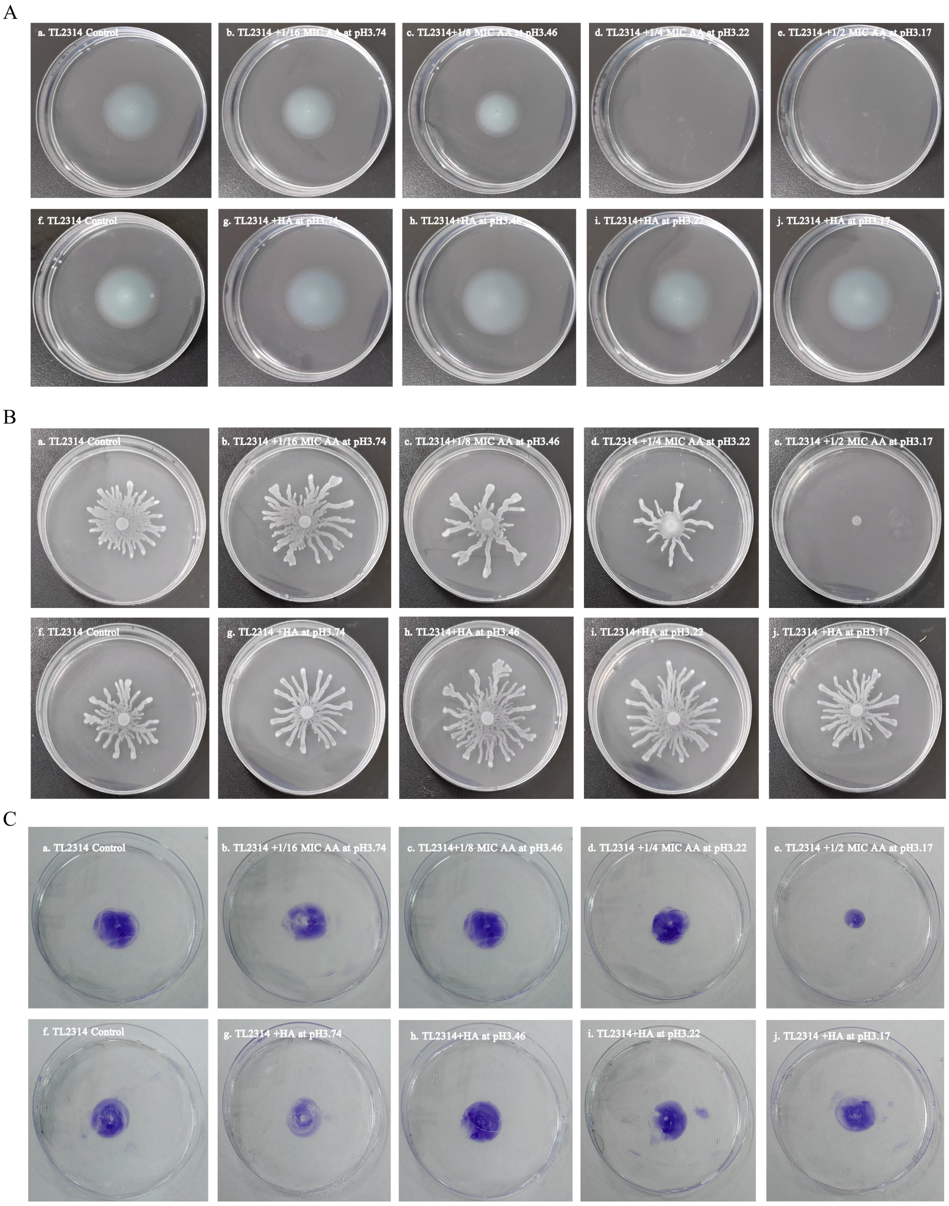

Figure S2 Effect of AA at subinhibitory concentrations (1/2×, 1/4×, 1/8×, 1/16× MICs) and no AA (control) on the motility against *P. aeruginosa* TL2314. (A) Effect of AA and HA corresponding to the pH of each concentration of AA on swimming activity of TL2314 . (a) TL2314 treated with the LB broth control; (b-e) TL2314 treated with 1/16×MIC, 1/8× MIC, 1/4× MIC, 1/2× MIC AA, respectively; (f) TL2314 treated with the LB broth control; (g-j) TL2314 exposed to HA corresponding to the pH of 1/16× MIC, 1/8× MIC, 1/4× MIC, 1/2× MIC AA, respectively. (B) Effect of AA and HA corresponding to the pH of each concentration of AA on swarming activity of TL2314. (a) TL2314 treated with the LB broth control; (b-e) TL2314 treated with 1/16×MIC, 1/8× MIC, 1/4× MIC, 1/2× MIC AA, respectively; (f) TL2314 treated with the LB broth control; (g-j) TL2314 exposed to HA corresponding to the pH of 1/16× MIC, 1/8× MIC, 1/4× MIC, 1/2× MIC AA, respectively. (C)

Effect of AA and HA corresponding to the pH of each concentration of AA on twitching activity of TL2314. (a) TL2314 treated with the LB broth control; (b-e) TL2314 treated with 1/16×MIC, 1/8×MIC, 1/4× MIC, 1/2× MIC AA, respectively; (f) TL2314 treated with the LB broth control; (g-j) TL2314 exposed to HA corresponding to the pH of 1/16× MIC, 1/8× MIC, 1/4× MIC, 1/2× MIC AA, respectively.

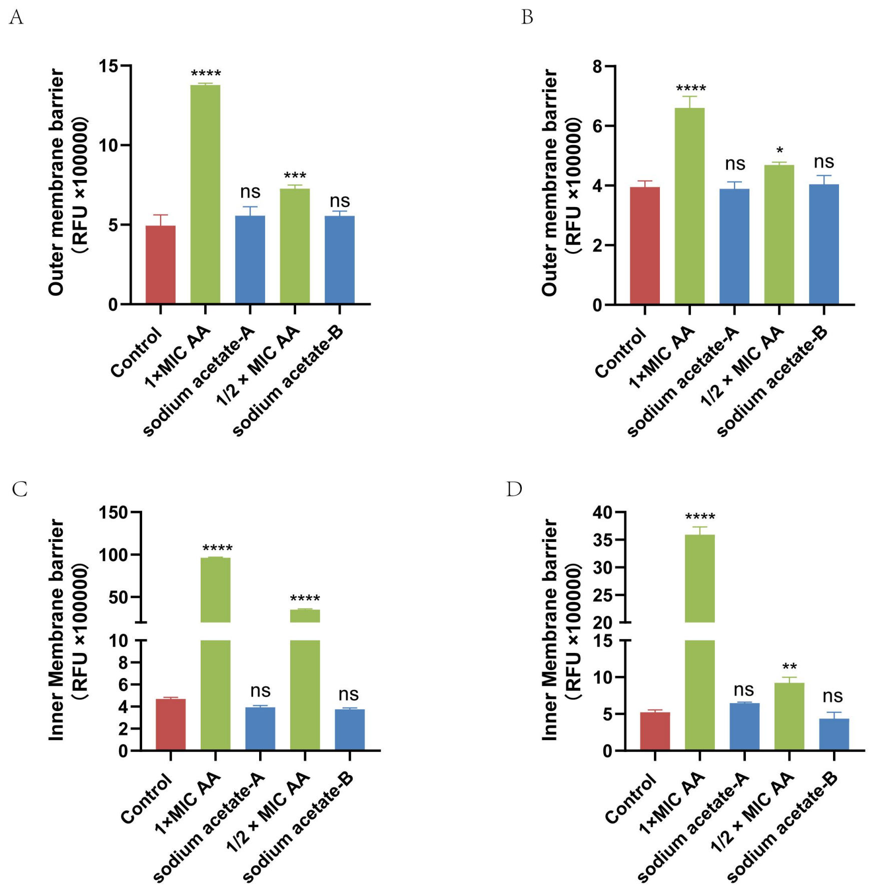

Figure S3 The potential antibacterial mechanism of AA. (A, B) Effects of AA and sodium acetate corresponding to each concentration of AA on the outer membrane permeability of TL1671 and TL2314, respectively; (C, D) Effects of AA and sodium acetate corresponding to each concentration of AA on the inner membrane permeability of TL1671 and TL2314 by using a plate reader, respectively; Sodium acetate-A, sodium acetate corresponding to 1×MIC AA; Sodium acetate-B, sodium acetate corresponding to 1/2×MIC AA. Data were analyzed by Student's t-test; ns, not statistically significant; $*P < 0.05$; $**P < 0.01$; $***P < 0.001$;$****P < 0.0001$. The experiments were performed thrice. The data are expressed as the mean ±standard deviation.

Table S1 The antimicrobial susceptibility of colistin and AA against COL-R *P. aeruginosa*

Strains	Strains source	Resistance determinants	MIC	
			COL (μg/ml)	AA (v/v)
ATCC 27853	reference	reference	0.5	0.078%
TL 1671	trauma seepage	PmrB (V15I, P216S)	32	0.078%
TL 1736	sputum	PmrB (V185A)	8	0.039%
TL 1744	sputum	PmrB (V15I, G68S)	4	0.039%
TL 2314	sputum	PhoQ (V260G)	8	0.039%
TL 2917	sputum	PmrB (G179D)<br>PhoQ (V260G)	8	0.039%
TL 2967	sputum	PmrB (D45E)	4	0.039%
TL 3008	sputum	PmrB (A190G)	4	0.039%
TL 3086	sputum	PmrB (S27R)	16	0.039%

Table S2. Primers used for qRT-PCR

Gene	Primer	Sequence (5′→3′)	Reference
rpsL	Forward	GCAAGCGCATGGTCGACAAGA	[36]
	Reverse	CGCTGTGCTCTTGCAGGTTGTGA	[36]
lasR	Forward	ACGCTCAAGTGGAAAATTGG	[35]
	Reverse	TCGTAGTCCTGGCTGTCCTT	[35]
rhlR	Forward	CATCCGATGCTGATGTCCAACC	[35]
	Reverse	ATGATGGCGATTTCCCCGGAAC	[35]
rhlI	Forward	AAGGACGTCTTCGCCTACCT	[35]
	Reverse	GCAGGCTGGACCAGAATATC	[35]
rhlA	Forward	GGCGATCGGCCATCT	[35]
	Reverse	AGCGAAGCCATGTGCTGAT	[35]
pqsA	Forward	GACCGGCTGTATTCGATTC	[37]
	Reverse	GCTGAACCAGGGAAAGAAC	[37]
exoT	Forward	TCTGCCGCCGAGATCAAGCA	[37]
	Reverse	TGTGATCCTTCGCCAGCCTCTC	[37]
exoS	Forward	CTCTACACCGGCATTCACTA	[37]
	Reverse	CTTCACTACCTGTTCAGCCT	[37]
exsA	Forward	CTGGCGAGTTGCTTTTCGTC	[37]
	Reverse	ACGCTCGACTTCACTCAACA	[37]
exoY	Forward	TGCCATAGAATCCGTCCTC–3	[38]
	Reverse	GATGACCGCCGATTATGAC	[38]
fliC	Forward	CTGACCTCGGTGCTGTTCAG	[37]
	Reverse	GAGCGTTGGTAGCGTTTTCC	[37]
